# A Nanoliposomal Gel Containing *Cinnamomum zeylanicum* Essential Oil with Effective Repellent against the Main Malaria Vector *Anopheles stephensi*

**DOI:** 10.1155/2022/1645485

**Published:** 2022-06-22

**Authors:** Mahmoud Osanloo, Samira Firoozian, Elham Zarenezhad, Zahra Montaseri, Saha Satvati

**Affiliations:** ^1^Department of Medical Nanotechnology, School of Advanced Technologies in Medicine, Fasa University of Medical Sciences, Fasa, Iran; ^2^Urmia Health Center, Disease Control Unit, Urmia University of Medical Sciences, Urmia, Iran; ^3^Noncommunicable Diseases Research Center, Fasa University of Medical Sciences, Fasa, Iran; ^4^Department of Infectious Diseases, School of Medicine, Fasa University of Medical Sciences, Fasa, Iran; ^5^Department of Medical Biotechnology, School of Advanced Technologies in Medicine, Medicine, Fasa University of Medical Sciences, Fasa, Iran

## Abstract

Malaria is the most important vector-borne disease; however, mosquito repellents are still a practical approach for controlling malaria, especially in endemic regions. Due to the side effects of synthetic repellents such as *N*, *N*-diethyl-meta-toluamide (DEET), the development of natural repellents has received much attention. In this study, nanoliposomes containing 0.5 and 2.5% w/v *Cinnamomum zeylanicum* essential oil were firstly prepared with particle sizes of 119 ± 6 and 195 ± 9 nm. Their morphologies and loading of the essential oil in the particles were then investigated using transmission electron microscopy (TEM) and attenuated total reflection-Fourier transform infrared (ATR-FTIR) analyses. The nanoliposomes were finally jellified to increase their viscosity and facilitate topical usage. The complete protection time of the nanoliposomal gel containing 2.5% *C. zeylanicum* essential oil was significantly longer than that of 2.5% DEET against *Anopheles stephensi*: 303 ± 10 > 242 ± 12 min, *p* < 0.001. Moreover, the prepared nanoformulation was stable for at least six months at 4 and 26°C. Therefore, the prepared prototype could be considered a natural repellent against the main malaria mosquito vector in field conditions. In addition, it is suggested to be investigated against other important factors mosquitoes.

## 1. Introduction

Human malaria is still the most important parasitic disease globally; its transmission occurs in all six regions of the World Health Organization (WHO). For instance, 324 million people are at risk in the Eastern Mediterranean region (EMRO) [1]. *Anopheles* mosquitoes transmit various *Plasmodiums* species such as *falciparum* and *vivax* that cause malaria in humans [2]. *Anopheles stephensi,* Liston, is one of the most important malarial vectors in the Middle East and South Asia [3, 4]. It has recently expanded to new parts of Ethiopia, Djibouti, Lakshadweep, and Sri Lanka. In addition, the spread of the species to Sudan, Somalia, Laos, Cambodia, Vietnam, and the Maldives has also raised concerns among public health officials [5, 6].

Insecticide-treated nets (ITNs) effectively control malaria in children and adults living in areas with persistent malaria transmission. Residual spraying (IRS) and space spraying of chemical insecticides are also recommended. However, in endemic regions and regions where vectors are exophilic and exophagic, repellents are recommended [7, 8]. DEET is the most common insect repellent; however, studies have reported that it irritates the eyes or skin and has neurological side effects [9]. In addition, when a repellent is exposed to the skin, about 3–19% of them may be absorbed by the body, leading to gastrointestinal side effects [10, 11]. Therefore, the development of new natural repellents is crucial.

Essential oils (EOs) with a wide range of biological effects, such as larvicidal, repellent, and adulticides, have received more attention [12, 13]. However, their efficacies should be improved. In recent years, formulating EOs in nanoformulations has proposed a practical solution to meeting the challenges [14–16]. The nanoliposomes are microscopic vesicles that closely resemble a cell membrane's structure and could carry different materials in their walls or interiors [17, 18]. In addition, features such as low intrinsic toxicity, biodegradability, amphipathic properties (hydrophilic and lipophilic), and lack of immunogenicity have made nanoliposomes an important carrier [19, 20].

In our previous study, the repellent effects of chitosan nanoparticles containing *Cinnamomum zeylanicum* EO (0.5%) were investigated; however, the efficacy was insufficient (31 min) [21]. Therefore, this study attempted to lengthen the complete protection time (CPT) by preparing the nanoliposomal gel containing two amounts of the *C. zeylanicum* EO (0.5 and 2.5%).

## 2. Materials and Methods

### 2.1. Materials

The *C. zeylanicum* EO was purchased from Green Plant of Life Pharmaceutical Co., Iran. Carboxymethyl cellulose (CMC), egg yolk lecithin, wool fat cholesterol, and absolute ethanol were purchased from Merck Chemicals Co., Germany. DEET 40% was purchased from Reyhan Naghsh Jahan Pharmaceutical Co., Iran. It was diluted to 2.5% using ethanol.

Five to seven-day-old unfed female *An. stephensi* mosquitoes were used in repellent tests. *An. stephensi* mosquitoes were reared at 27 ± 2°C (by using the electric heater), 70% relative humidity (by using the evaporator), and D12 : L12 light period (by using the lamp equipped with on/off timer) in the insectary of Urmia University of Medical Sciences, Iran. The mosquitoes were not in contact with any insecticide and were artificially fed with blood to lay eggs; defibrinated sheep's blood was poured on glass and drawn with a parafilm and was placed upside down on the cage. A container of hot water was placed on it to adjust the temperature; thus, artificial blood-feeding was performed. In addition to two blood meals each week, 10% sugar or honey water solutions were used to feed the adults. Forty-eight hours after feeding the mosquitoes, two-third of a small porcelain bowl (5 × 12 cm) was filled with clean water and was placed in the mosquito cage to allow for oviposition. The eggs were transferred to larval containers and fed with fish food. After the emergence of the required population, they were used for repellent tests.

### 2.2. Preparation and Characterizations of Nanoliposomal Gels Containing the *C. zeylanicum* EO

**Figure 1:**
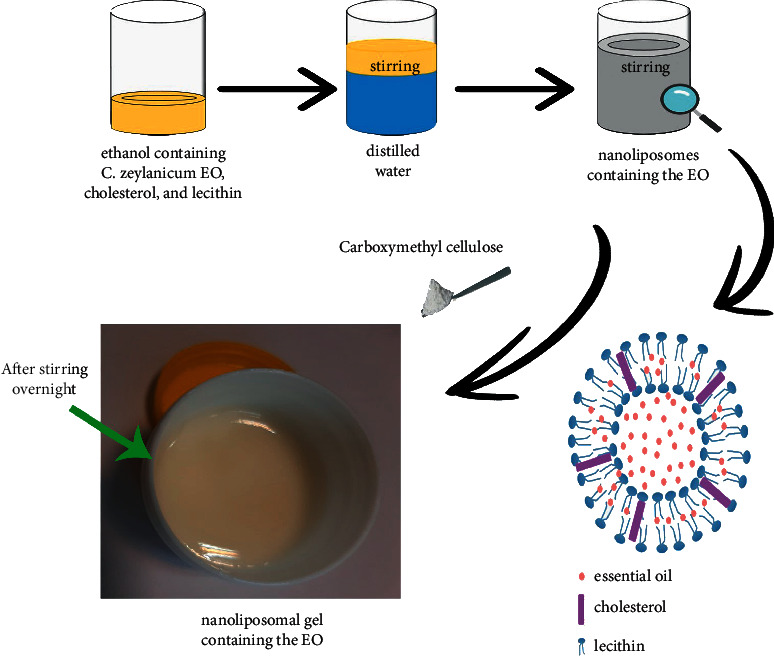
Preparation of the nanoliposomal gel containing *C. zeylanicum* EOs.

Nanoliposomes containing 0.5 and 2.5% *C. zeylanicum* EOs were prepared using the ethanol injection method. Lecithin, cholesterol, and *C. zeylanicum* EO (at two amounts) were first dissolved at proper amounts in absolute ethanol (2000 rpm, room temperature, and overnight). Then, one mL of the obtained solution was added dropwise to 4 mL of distilled water; the concentrations of lecithin and cholesterol were fixed at 3 and 1% w/v, and the EO was fixed at 0.5 and 2.5% w/v. Finally, the mixture was stirred for 40 minutes to stabilize the formed nanoliposomes (Figure 1).

The particle size of the nanoliposomes was investigated by a dynamic light scattering type aperture (K-One Nano, Ltd, Korea). Particle size distribution was also calculated in the equation; *d*90 − *d*10/*d*50, where *d* is the diameter and 90, 10, and 50 are percentiles of the particles with a lower diameter than these values.

The attenuated total reflection-Fourier transform infrared (ATR-FTIR) analysis investigated the successful loading of the EO into nanoliposomes. The nanoliposomes and free nanoliposomes (without EO) were first centrifuged for 60 min at 12000 g (4°C). Then, the obtained pellets were kept at room temperature for three days to reduce their moisture and subject to the instrument (Bruker Company, Model Tensor II, USA). The spectra were recorded in the range of 400–4000 cm^−1^.

Furthermore, CMC as a thickening agent was added to the prepared free/nanoliposomes containing 0.5 and 2.5% EOs to increase the viscosity and facilitate topical use. The prepared gels were named LipoGel 0.0%, LipoGel 0.5%, and LipoGel 2.5%, respectively; they were considered for stability and repellent tests. The gels were held at two temperatures (4 and 26°C) for six months and checked for sedimentation, cremation, and biphasic.

### 2.3. Repellent Assay

Repellent assays were performed under insectarium conditions using the WHO recommended guideline for the arm-in-cage method with a slight modification [22]. The complete protection time (CPT) as the outcome is defined as the interval between the samples' topical application (LiopGel 0.0, 0.5, and 2.5% and DEET 2.5%) and the first mosquito landing.

The volunteers' hands were washed with odorless soap and water, which was soaked with 70% alcohol, and dried. Nor did they smoke or use perfume, cream, and lotion for 12 hours before. Fingers up to the wrists were protected with odorless latex gloves to prevent bites; 25 cm of the untreated forearm skin (wrist to elbow) was exposed to mosquitoes. The tests were repeated three times, and CPTs were reported as the mean ± standard deviation. The final values for all samples were compared with one-way ANOVA using SPSS software (v. 22).

For testing, cubic wooden cages 40 × 40 × 40 cm were used; they were covered with a fine mesh and had a cloth sleeve at the front. The treated volunteers' forearms were placed (motionless) in a cage containing 200 hungry female mosquitoes (without access to blood and sugar water) for 3 min to ensure that the mosquitoes were motivated to land and bite. The same procedure was then consistently repeated at every half-hour interval. However, after the first repetition and estimation of the CPT, exposure to mosquitoes was continuously carried out for a final 30 min.

## 3. Results

### 3.1. Particle Size and Particle Size Distribution of the Nanoliposomes

**Figure 2:**
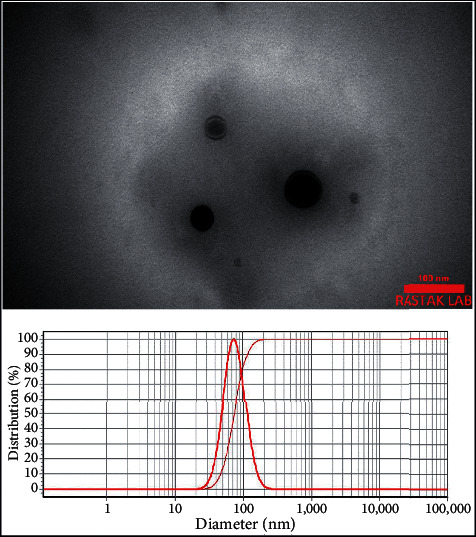
TEM and DLS analyses of nanoliposomes containing the 0.5% *C. zeylanicum* EO with a particle size of 119 ± 6 nm (SPAN 0.98).

**Figure 3:**
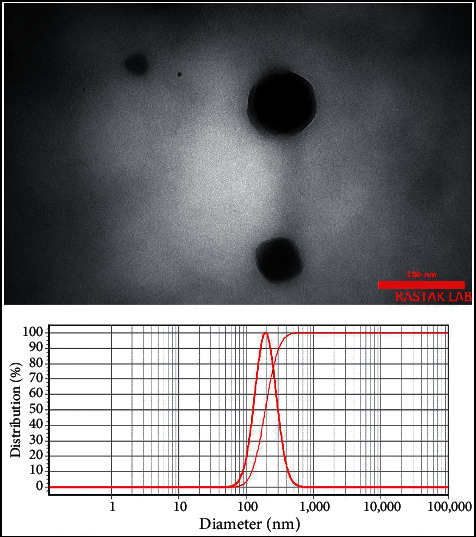
TEM and DLS analyses of nanoliposomes containing the 2.5% *C. zeylanicum* EO with a particle size of 195 ± 9 nm (SPAN 0.96).

TEM images and DLS profiles of the nanoliposomes containing 0.5 and 2.5% *C. zeylanicum* EOs with 119 ± 6 and 195 ± 9 nm sizes are depicted in Figures 2 and 3. The distributions of the particles were calculated 0.98 and 0.96, respectively, possessing narrow particle size distribution as values that were lower than 1 [23]. Besides, both particles were spherical.

### 3.2. Physicochemical Properties of the Nanoliposomal Gel

No sedimentation, cremation, or phase separations were observed after six months of storage of the gels (LipoGel 0.0%, LipoGel 0.5%, and LipoGel 2.5%) at two temperatures (4 and 26°C).

**Figure 4:**
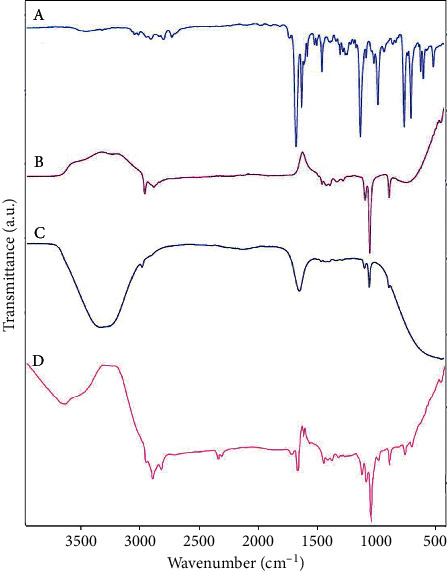
ATR-FTIR of A: *C. zeylanicum* EO, B: free liposomes, C: nanoliposomes containing the *C. zeylanicum* EO, and D: nanoliposomes gel containing the *C. zeylanicum* EO.

The ATR-FTIR spectra of *C. zeylanicum* EOs, free liposomes, nanoliposome containing EO, and the nanoliposomal gel containing EO are shown in Figure 4 (A, B, C, and D). The spectrum of *C. zeylanicum* EOs displays a broad peak at 3468 cm^−1^; it is allocated to OH related to alcohol and phenols in EO. The bands at 3061 and 3028 cm^−1^ relate to =C-H, the band at 2923 cm^−1^ shows –CH stretching vibration of alkanes, and the bands at 2812 and 2740 cm^−1^ indicate C-H aldehyde. Besides, the strong peak at 1728 cm^−1^ represents the aldehyde of saturated fat, and the peaks at 1671 and 1624 cm^−1^ correspond to the stretching vibration of an aldehyde carbonyl C=O bond. These main and strong peaks revealed a high amount of cinnamaldehyde and aldehydes in the *C. zeylanicum* EO, which agrees with the GC-MS analysis report of the used EO [24].

The ATR-FTIR spectrum of free liposomes showed the bands at 2981 and 2904 cm^−1^ attributed to C-C-H stretching, the band at 1454 cm^−1^ displays CH_2_ bending, and the characteristic absorption at around 1385 cm^−1^ showed CH_3_ bending. The band at 1274 cm^−1^ demonstrated the presence of the P=O group in lecithin. The strong peak at 1044 cm^−1^ relates to C-O stretching.

The ATR-FTIR spectrum of nanoliposomes containing the *C. zeylanicum* EO confirmed that the EO successfully loaded in the nanoliposome wall. The strong and broadband at 3000 to 3500 cm^−1^ related to OH due to intermolecular hydrogen bonding showed the EO encapsulated in nanoliposomes. The sharp and new peak at 1639 cm^−1^ shows C=O in the EO. The very weak band at 1270 cm^−1^ is related to the P=O group; however, it was lower in the spectra of nanoliposomes containing the EO than in free liposomes due to limitation in the motion of the P=O group that interacts with the EO. All the other absorption bands appear in the spectra of the free liposome.

The ATR-FTIR spectrum of LipoGel showed the broadband at about 3200–3600 cm^−1^attributed to the hydroxyl group due to hydrogen bonding. The bands at around 2924 and 2853 cm^−1^ correspond to C-H stretching due to the *C. zeylanicum* EO and CMC. The characteristic band at about 2359 and 2342 cm^−1^ can be related to COO^−^ and OH bending in CMC. The peak at about 1731 and 1674 cm^−1^ can be related to C=O stretching representing carbonyl groups in the *C. zeylanicum* EO and CMC. The weak peak at about 1575 cm^−1^ displayed carboxylate groups stretching vibrations. The band at about 1272 cm^−1^ related to the presence of the P=O group. The sharp and strong peak at 1044 cm^−1^ demonstrated C-O stretching.

### 3.3. Repellent Effects of the Nanoliposomal Gels Containing the *C. zeylanicum* EO

**Figure 5:**
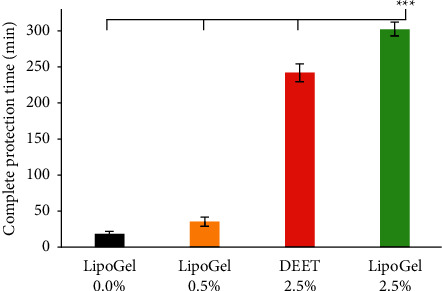
Complete protection time of the samples against *An. stephensi.*

In Figure 5, LipoGel 2.5% with CPT 303 ± 10 min was substantially more potent than all samples (*p* < 0.001). The second high-efficacy sample was 2.5% DEET with CPT 242 ± 12. Besides, LipoGel 0.5% with CPT 35 ± 6 min was significantly more potent than LipoGel 0.0% with CPT 18 ± 4 min (*p*=0.015).

## 4. Discussion

The chemical composition of the used *C. zeylanicum* EO was investigated using gas chromatography-mass Spectrometry and reported in our previous study [24]. As a result, cinnamaldehyde (62%), linalool (7%), transcaryophyllene (7%), transcinnamyl acetate (4%), and benzyl benzoate (3%) were identified as five major compounds [24].

In the current study, nanoliposomes containing the *C. zeylanicum* EO were first prepared using the ethanol injection method. Due to the high solubility of fats in ethanol, the preparation of nanoliposomes containing higher amounts of EOs than polymeric nanoparticles is possible in this method [25, 26]. Besides, due to the hydrophobicity of EOs, nanoliposomes have a higher loading capacity than chitosan nanoparticles [27, 28]. However, due to the low viscosity of the prepared nanoliposomes, its topical application was a challenge; by adding a thickening agent and transforming them into a gel, the challenge was thus met. Interestingly, the nanoliposomal gel containing the 2.5% *C. zeylanicum* EO with CPT 303 min was achieved under the required condition of 2 h CPT of the Environmental Protection Agency regulation to register as a repellent [29].

Furthermore, some reports on using other nanoformulations as mosquito repellents have been published, e.g., solid-lipid nanoparticles containing *Zataria multiflora* (1%) with CPT 95 min against *An. stephensi* [30]. In another work, chitosan nanoparticles containing the *C. zeylanicum* EO (0.5%) were applied against *An. stephensi* with a CPT of 31 min [21]. Another paper reported protection times of nanoemulsion containing *Mentha piperita* and *Eucalyptus globulus* EOs at 4.17 h and 5.51 h against *An. stephensi* [31]. Noted, those nanoformulations contain 50% EOs, so achieving high protection time is predictable; however, to commercialize such nanoformulations, which contain large amounts of EOs, their pungent odor must be manipulated. Another research reported protection times nanosized microemulsions containing 5, 10, and 15% *Eucalyptus globulus* EOs with 82, 135, and 170 min [32]. In the mentioned research, a mixture of mosquitoes was used: *Culex pipiens* (62%), *Ochlerotatus caspius* (22%), *Culex pusillus* (10%), and *Culex tritaeniorhynchus* (6%).

## 5. Conclusion

The nanoliposomal gel containing 0.5 and 2.5% *C. zeylanicum* EOs and the free liposomal gel (LipoGel 0.0%) were prepared. The complete protection times of them and DEET (2.5%) were investigated against *An. stephensi* using the arm-in-cage method. LipoGel 0.0% did not show proper efficacy (18 ± 4 min); however, the gel containing the 2.5% EO (LipoGel 2.5%) was significantly more potent than DEET 2.5%. Therefore, it could be considered for further investigations under field conditions and other important mosquitoes.

## Data Availability

The data generated or analyzed during this study are available from the corresponding author on reasonable request.
